# Decision Support Systems adoption in pesticide management

**DOI:** 10.12688/openreseurope.17577.2

**Published:** 2024-09-26

**Authors:** Jotham Jea Akaka, Aurora García-Gallego, Nikolaos Georgantzis, Jean-Christian Tisserand, Efi Vasileiou, Mark Ramsden

**Affiliations:** 1 Laboratori d'Economia Experimental and Department of Economics, Universitat Jaume I, Castellón de la Plana, Valencian Community, 12071, Spain; 2 Centre de Recherche sur les Entreprises (CEREN), Burgundy School of Business, Dijon, France; 3 City College, European Campus, University of York, Thessaloniki, Greece; 4 RSK ADAS, Cheshire, UK

**Keywords:** Agriculture, Decision Support Systems, Integrated Pest Management

## Abstract

This paper presents the findings from a survey on factors influencing the adoption of agricultural Decision Support Systems (DSS). Our study focuses on examining the influence of behavioural, socioeconomic and farm specific characteristics on DSS adoption. Using two structural equation models, we investigate how these factors influence the willingness to pay (WTP) and willingness to adopt. Our analysis reveals nuanced insights into the user and farm-specific factors that influence the decision-making process of DSS adoption and WTP. Notably, farm size significantly influences both adoption and WTP, with larger farms more likely to adopt and exhibit higher WTP. To promote adoption, it is important to adapt promotion strategies, with a focus on productivity benefits for large-scale farms and addressing price barriers for smaller ones. Additionally, the main crop type grown impacts WTP and adoption, with arable crop farmers exhibit a lower WTP but more likely to adopt, especially in large-scale operations. Conversely, small-scale arable farmers exhibit higher WTP but lower adoption rates due to scale constraints. Farmer characteristics such as experience and attitudes also play a crucial role, with experienced users and those perceiving productivity improvements due to DSS showing higher WTP. In addition, adoption is also influenced by ease of use and pricing, underpinning the importance of user-friendly designs and clear cost justifications. DSSs with user-centric designs and clear cost justifications can enhance adoption rates.

## Introduction

Invertebrate pests, pathogens and weeds (collectively ‘pests’) can reduce both the quantity and/or quality of crops and require continuous management. According to the EU’s Sustainable Use Directive (SUD 2009/128/EC) and specifically the third principle of Integrated Pest Management (IPM), farmers' decision making should be supported using forecasts, economic thresholds or other IPM DSSs (Intergrated Pest Management Decision Support Systems) ( Barzman *et al*., 2015). Despite many IPM DSS being available in the agricultural sector, including some examples of well tested and implemented systems ( Shtienberg, 2013), uptake by farmers and their advisers remains low ( Akaka *et al*., 2023; Lázaro *et al*., 2021). Uptake has been constrained by numerous factors, associated with either the technical development and availability of systems, or the level of engagement between various stakeholders during development and product launch. The latter frequently results in DSS being either rejected by potential users, or the outputs of the system are not perceived as sufficiently trustworthy to influence pest management decisions.

DSSs provide farmers with reliable scientific data, promote innovation, and reduce wastage ( Fountas *et al*., 2005; McCown *et al*., 2009; Thorburn *et al*., 2011). These systems use various data sources such as soil data and pest population dynamics to align pesticide application with actual crop risk. In fact, the use of DSSs has been shown to reduce fungicide application by 50% without increasing pesticide use related risk ( Lázaro *et al*., 2021). Consequently, this targeted approach reduces environmental, economic and pesticide resistance risks.

The agricultural sector faces the challenge of a growing global demand for food, whilst preserving biodiversity, combating climate change and ensuring that production is sustainable and economical ( Aubert *et al*., 2012). The decisions associated with pest management can be complex ( Ramsden *et al*., 2017). Applying pesticides when the actual risk of pest damage is low rarely leads to acute repercussions but has a profound impact on the long-term sustainability of crop production and the health of the surrounding ecosystem ( Aktar *et al*., 2009). Conversely, farmers can experience substantial economic losses when pest infestations are left unchecked and cause widespread crop damage ( Oliveira *et al*., 2014). The use of DSSs in agriculture can significantly reduce pesticide related risk by leveraging data driven insights towards optimal timing and application of pesticides.

The main purpose of a DSS is to aid users to make effective decisions. For the outputs to be trusted, DSSs must not only be reliable, but also clear in their limitations and assumptions ( Rossi *et al*., 2014). Currently, the common types of IPM DSS used are tactical systems, supporting decisions on when to monitor crops for pest activity and when action may be required to prevent economic losses, such as monitoring systems, forecasting systems and thresholds ( Damos, 2015). Monitoring systems report actual occurrences of given pests at a defined spatial scale. Pest forecasts are based on algorithms that model certain pest ecology from climatic and/or agronomic inputs to predict the likelihood of infestation and/or the crop risk ( de la Fuente *et al*., 2018). Together, they provide insight into the present and future risk of infestation in their region, supporting decisions on whether further crop monitoring and/or management actions are required ( Zhiembayev *et al*., 2020). Economic thresholds are based on the potential for a given pest to cause economic damage to the crop and are a final assessment for action ( Ramsden *et al*., 2017). A majority of IPM DSS require user inputs such as field location, soil type and structure, climate data, and agronomic details such as sowing date and crop variety. Based on this data, the DSSs algorithms are run, and the system outputs potential strategies (decision aids/treatments) ( Seem & Russo, 1984). Outputs range from a relative assessment of the risk of infestation and/or economic damage to specific recommendations for treatment, such as application of a given pesticide or bioprotectant.

Over the last three decades, there is a growing concern that even though agricultural DSSs are available to farmers, they are neither fully utilised nor appropriate for the complexity of the daily decisions farmers face ( Eastwood *et al*., 2012; Van Meensel *et al*., 2012a). In part, this may be due to a lack of evidence that the use of DSS leads to improved economic returns, and there is increasing effort to address this. In weed management, for example, some DSSs determine the density of weeds at which a control treatment will provide an economic return ( Yuan *et al*., 2023). Economic thresholds such as this are highly valued by farmers and have been developed for many pests ( Auld & Tisdell, 1987; Neeser *et al*., 2004), albeit difficult to develop and incorporate in routine farming practice ( Ramsden *et al*., 2017).

From inception, most DSSs are primarily designed to handle farm and crop production data. The weakness of this approach is that the role of the farmer, and other auxiliary factors, are minimised or left out completely. User characteristics are, however, key factors influencing the adoption and application of DSS ( Aubert *et al*., 2012). The data-focused nature of DSS is an understandable by-product of developers' efforts to optimise their DSS to work with certain types of pests and crops, making them specific and detailed. In contrast, farmers prefer to have a broader view, reflecting the polyvalent spectrum of day-to-day decisions they must make ( Rossi *et al*., 2014). Van Meensel *et al*. (2012b) conclude that not only are some DSS are overwhelmingly complex, but also the terminology and functions are not only unsuitable but often irrelevant to the end user ( Kutter *et al*., 2011). Other DSS may be simple, but the Graphical User Interface (GUI) is too complex, either by poor design, or due to the high frequency of changes made by developers to optimise their product ( Van Meensel *et al*., 2012b). Therefore, if farmers interact with a few complex systems, it can lead to a community level perception of all DSS being too complicated to be of value. In addition, many DSSs lack a proven track record of their effectiveness. This is in part explained by the DSS development cycle, in which systems are a culmination of a short term (3–5 year) research project ( Rahn, 2004). Often, the main activities involve coding and GUI design, instead of demonstrating the economic and environmental benefits the system offers. A focus on algorithm development, and optimal GUI design, with omission of the influence of user behaviours has led to dismal adoption and a general lack of trust in agricultural DSS among farmers.

Decision making and technological adoption theories provide useful insights into the low rates of adoption of agricultural DSSs. The theories of adoption of farming practices have been extensively documented, identifying the factors influencing the adoption of farming technology ( Feder & Umali, 1993; Kuehne *et al*., 2017). Conventionally, decision-making process theories identify three main factors: farmer, technological and institutional characteristics ( Meijer *et al*., 2015). A review by Kumar, Engle & Tucker (2018) identified the source of information, technological features, economic factors, farm characteristics, institutional and sociodemographic factors significantly influencing the adoption of farming technologies. A paper by Akaka *et al*. (2023) identified farm size, the type of farm, and the farmer’s willingness to pay as significant predictors of DSS adoption.

As suggested by Davis (1989) in the Technology Acceptance Model, technology acceptance and usage are determined by the perceived usefulness and the perceived ease of use. Perceived usefulness is the utility gained from improving one’s work performance due to the use of a certain technology, whereas perceived ease of use is related to the effort required to use a certain technology ( McDonald *et al*., 2016)

The concepts of willingness to adopt and WTP for the use of agricultural DSSs are closely related but often misunderstood. It is possible to have farmers who are willing to adopt but not willing to pay. Agriculture often involves the exploitation of public goods that have a monetary value difficult to quantify ( Hoekstra, 2013). Thus, even if some farmers are well informed on the benefits of IPM DSS, they might be less willing to pay due to their subjective valuation of the relative importance of public goods.

Even though the investigation into the factors influencing adoption of agricultural DSS is vast, a majority of this research mostly identified technological features and user traits (age, gender, education, income) as the main factors that influence the adoption of DSSs. However, the influence of other adoption related factors such as experience, individual risk attitudes, trust and their willingness to pay to use DSSs has received little attention. There is still a noticeable gap in comprehensively analysing the interaction between individual attitudes, trust, risk attitudes, individual experience among other factors in the use and monetary valuation of agricultural DSSs.

To address this gap, this paper investigates how socioeconomic factors, risk attitudes, trust and technological characteristics drive the willingness to pay and adoption of agricultural DSSs using structural equation modelling to explore the complex decision-making process behind adoption and WTP. In general, our analysis revealed a complex relationship between a set of interdependent factors that influence farmers' decision to adopt and their WTP for these systems. These factors fall into the broad categories of socioeconomic factors, farm characteristics, user attitudes, farmer experience, risk attitudes, trust and user engagement. To identify the key factors influencing WTP and willingness to adopt, the following research questions are formulated.

- What socioeconomic factors influence farmers' willingness to adopt and WTP agricultural DSSs?
- Do farmer attitudes influence adoption and WTP of agricultural DSS?
- How do farmers’ risk attitudes influence willingness to adopt agricultural DSSs?
- Does farmer trust influence WTP for agricultural DSSs?

The rest of this paper is structured as follows. Section 2 presents the specific hypotheses of our research questions and the methodology, followed by results in Section 3. Finally, the discussion and main conclusions are presented in Section 4.

## Survey to model Adoption decision and WTP

Based on the research questions presented above, a survey, which was pilot tested in the meetings of the PME of the IPM Decisions project among participating researchers and stakeholders, was conducted among farmers participating in workshops organised in the framework of the project IPM Decisions. The survey was designed to address several testable hypotheses, which we present below in detail under each of the two models estimated:

Model 1: Willingness to Adopt DSS

H1: Farm size is positively associated with adoption of DSS.

H1a: This positive association is stronger for farmers with higher income

H1b: This positive association is stronger among arable crop farmers

H2: Specialising in a single (unique) crop is positively associated with adoption

H2a: This effect is stronger among male farmers

H3: Farmers who enjoy using new technologies are more willing to adopt

H3a: This effect is stronger among younger farmers

H4: Farmers who believe that DSS will improve their productivity is positively associated with adoption

H5: Farmers who value ease of use as a DSS feature are more likely to adopt DSS

H6: Farmers who occasionally attend DSS demonstrations are more likely to adopt

H7: Compliance with legislative requirements is positively associated with adoption

Model 2: Willingness to Pay (WTP) for DSS

H1: Trust in DSS is positively associated with WTP

H1a: This effect is positively influenced by trust in others

H1b: Risk attitudes moderate the effect of trust in others on trust in DSSs

H1c: This effect is stronger among risk averse farmers

H1d: This effect is stronger among farmers with higher sensitivity to risk

H1e: This effect is stronger among farmers who regularly receive marketing information

H1f: This effect is stronger among farmers who are willing to try new products

H2: Low price as a DSS characteristic is positively associated with adoption.

H3: Larger farms is positively associated with higher WTP

H3a: This effect is stronger with higher farm income

H3b: The level of satisfaction with current production moderates this effect

H4: Arable crop farming is positively associated with WTP

H5: Past experience with DSS is positively associated with WTP

H5a: This effect is moderated by the belief that DSS improves productivity

H5b: This moderating effect is stronger among farmers who use the same DSS as their advisors

The questionnaire survey was administered to 149 farmers from 11 European countries ( Table 1), eliciting personal, farm and farm equipment characteristics that influence the adoption and trust in DSS. The survey was designed in consultation with a team of experts working closely with the IPM Decisions Network. The following are the countries that participated in the survey: Italy (37), United Kingdom (24), Greece (22), Slovenia (14), Sweden (11), Lithuania (9), Finland (9), Germany (9), France (6), Denmark (4) and Holland (4). The questionnaire was completed by farmers in face-to-face national workshops held between December 2019 and March 2020.

**Table 1.** Descriptive statistics (N=149).

Category	Variable	Obs	Mean	Std. Dev.	Min	Max
Dependent variables	DSS use	145	.779	.416	0	1
	Willingness to pay to use DSS	146	.5	.502	0	1
DSS characteristics	Low price	136	3.184	1.373	1	5
	Ease of use	140	4.379	1.076	1	5
External environment	Legislative requirements	138	.797	.404	0	1
Farmer characteristics	Age	149	2.067	.852	1	3
	Income	144	2.924	1.468	1	5
	Male	148	.811	.393	0	1
Farm characteristics	Arable crops	117	.829	.378	0	1
	Unique crop	113	.593	.493	0	1
	Farm size	148	3.108	1.114	1	4
Farmer experience	Already used DSS	126	.714	.454	0	1
	Use same DSS as advisor	109	.349	.479	0	1
Farmer attitudes	DSS productivity	147	.327	.471	0	1
	Enjoy using new technology	147	3.612	1.306	1	5
	Satisfaction with current production level	107	2.374	1.145	1	5
	Willingness to try new products	143	.811	.393	0	1
Risk attitudes	Average risk aversion	105	0	.958	-1.69	1.824
	Sensitivity to risk	105	0	.559	-1.916	1.508
Trust	Trust in colleagues' advice	148	2.736	1.084	1	5
	Trust in DSS	148	2.784	.993	1	5
	Trust in friend’s advice	147	2.707	1.16	1	5
User engagement	Marketing info about DSS	127	.047	.213	0	1
	Occasional DSS demonstrations	143	.49	.502	0	1

## Descriptive statistics

In line with the General Data Privacy Policy, respondents were free to exclude any information they did not wish to share but were encouraged to provide as much information as possible. All participants participated on site to the survey and no responses were excluded. No recording or other instruments were used, except for paper and pencil responses to the survey. Thus, there are missing observations for some variables resulting in an uneven dataset. A summary of the descriptive statistics is provided in Table 2. An overview of all the variables created from the questionnaire is provided in Table 1 (see Appendix A in Extended data).

According to Table 1, the subjects show a high likelihood of adoption (mean = .779) but neutral on their WTP to use DSSs (mean = .5). The average acreage of a farm is between 30–60 hectares (mean = 3.1) and a majority of the farmers specialise in arable crop farming (mean = .829). A significant proportion of farmers have prior experience with DSSs (mean = .714), whereas only 35% use the same DSS as their advisors. Even though farmers in this survey believe that DSS can improve their productivity (mean 3.27 on a 1–4 scale), their receptiveness to new technologies is slightly above neutral (mean = 3.612 on a 1–5 scale).

## Data analysis

The data is analysed using a partial least squares structural equation model (PLS-SEM) that is robust to small samples, non-normal distributions and suitable for modelling complex relationships. ( Hair *et al*., 2019). We use this approach to estimate two SEMs to identify factors influencing willingness to adopt DSS on one hand and WTP to use DSS on the other hand. The analysis was conducted in two stages using Stata version 14 ( StataCorp, 2014) and SmartPLS version 4 ( Ringle *et al*., 2022).

All the variables used in the analysis were checked to identify and control for the effect of extreme values present in the data matrix of the independent variable on the dependent variable. Positive cases of such distributions were remedied by collapsing the responses into fewer categories than in the original questionnaire. In addition, the reduction of the scale to fewer items than the original improves interpretability of the results. Before running the SEMs, we run factor analysis on the four highly correlated measures of risk attitudes from the panel lottery task (see question 42, Appendix D in Extended data) to construct two salient measures of risk aversion. This risk elicitation method offers a complete description of risk attitudes than single-task unidimensional risk elicitation methods and accounts for differences of actions across different risk-taking contexts. It consists of four panels of lotteries designed to compensate for lower probability of winning with a higher expected payoff ( Sabater-Grande & Georgantzis, 2002). Thus, the test exposes participants to a choice of the preferred lottery, thus eliciting 1) the respondent’s propensity to take riskier choices and 2) the respondent’s tendency to alter their decisions according to changes in the risk-return parameter. Since these measures are highly correlated, we use factor analysis to extract two salient components that account for more than 80% of the total variance and are uncorrelated (see appendix B, tables 1 and 2 in Extended data). The first component captures risk aversion due to the relatively balanced positive loading of the four panels, while the second component involves the difference of choices between panel 4 and 1 and between panel 3 and 2 (with the former difference being loaded twice as much as the latter).

In both SEMs, the analysis focuses on the measurement evaluation model and the relationships between the indicators and the constructs. This study uses only reflective measurement, because most of the indicator variables are made up of a single construct. The Cronbach’s alpha value for all multi-item constructs is greater than 0.7, confirming constructs’ internal consistency ( Hair *et al*., 2019). The Average Variance Extracted (AVE) for each multiple-item construct is higher than 0.5 for all groups, which corroborates convergent validity. Finally, to examine the discriminant validity, the heterotrait-monotrait ratio of correlations (HTMT) is applied. The estimated HTMT values are below the threshold of 0.9 for all the constructs ( Hair *et al*., 2019). Table 4 and 5 in Appendix B and Table 2 and 4 in Appendix C ( *Appendices for the Paper ‘Decision Support Systems Adoption in Pesticide Management’*, 2024.) in Extended data contain the VIF of the variables and R ^2 ^test statistics for multi-item constructs in the DSS, directly influencing adoption and WTP, respectively.

## Results

This section presents the results obtained from the PLS estimates for factors influencing adoption and factors influencing WTP respectively.

## Factors influencing adoption

**Figure 1.**
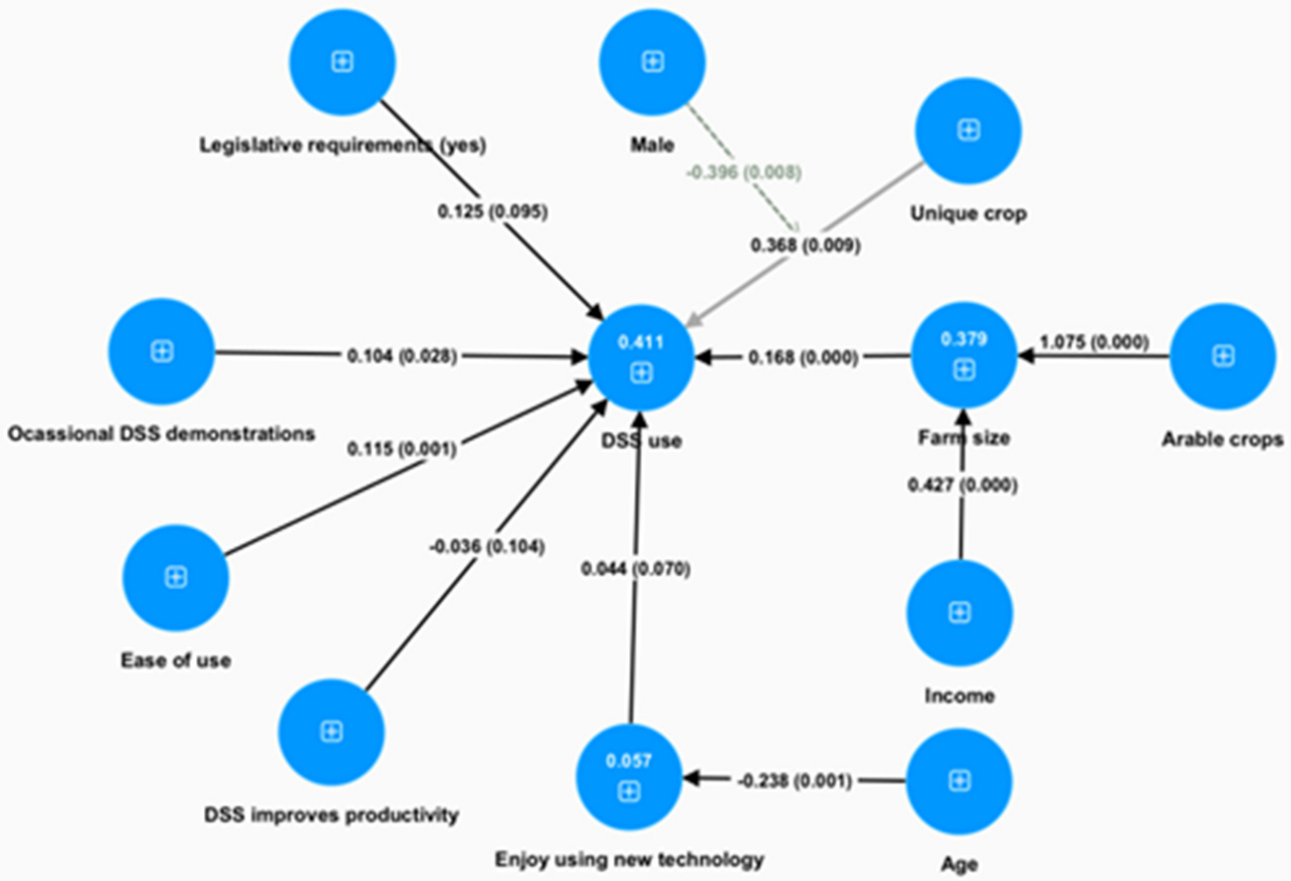
DSS adoption decision model (SEM estimated with SMART PLS). Numbers in circles are R ^2^ values, numbers on the arrows are path coefficients and in parentheses are the p-values.

Figure 1 presents the interconnections among variables which can explain the decision process leading to the adoption of DSS (complete results can be found in appendix B, table 3 in Extended data). Its effect is more pronounced by its two associated factors, income and arable crops. A unique crop managed by a farmer has also a positive impact. Both factors’ impact on DSS adoption is mediated by male gender: positively for farm size and negatively for unique crop. This is the only gender effect captured by our analysis. A young age is the other demographic trait that enhances the probability of adopting a DSS, and its effect emerges since younger users enjoy using new technology.

Among all positively perceived characteristics of a DSS, the only ones that matter in DSS adoption are the perception that the DSS is easy to use. Finally, the only external intervention that was found to significantly contribute to a farmer’s DSS adoption decision is exposure to demonstration sessions.

### Factors influencing WTP

**Figure 2.**
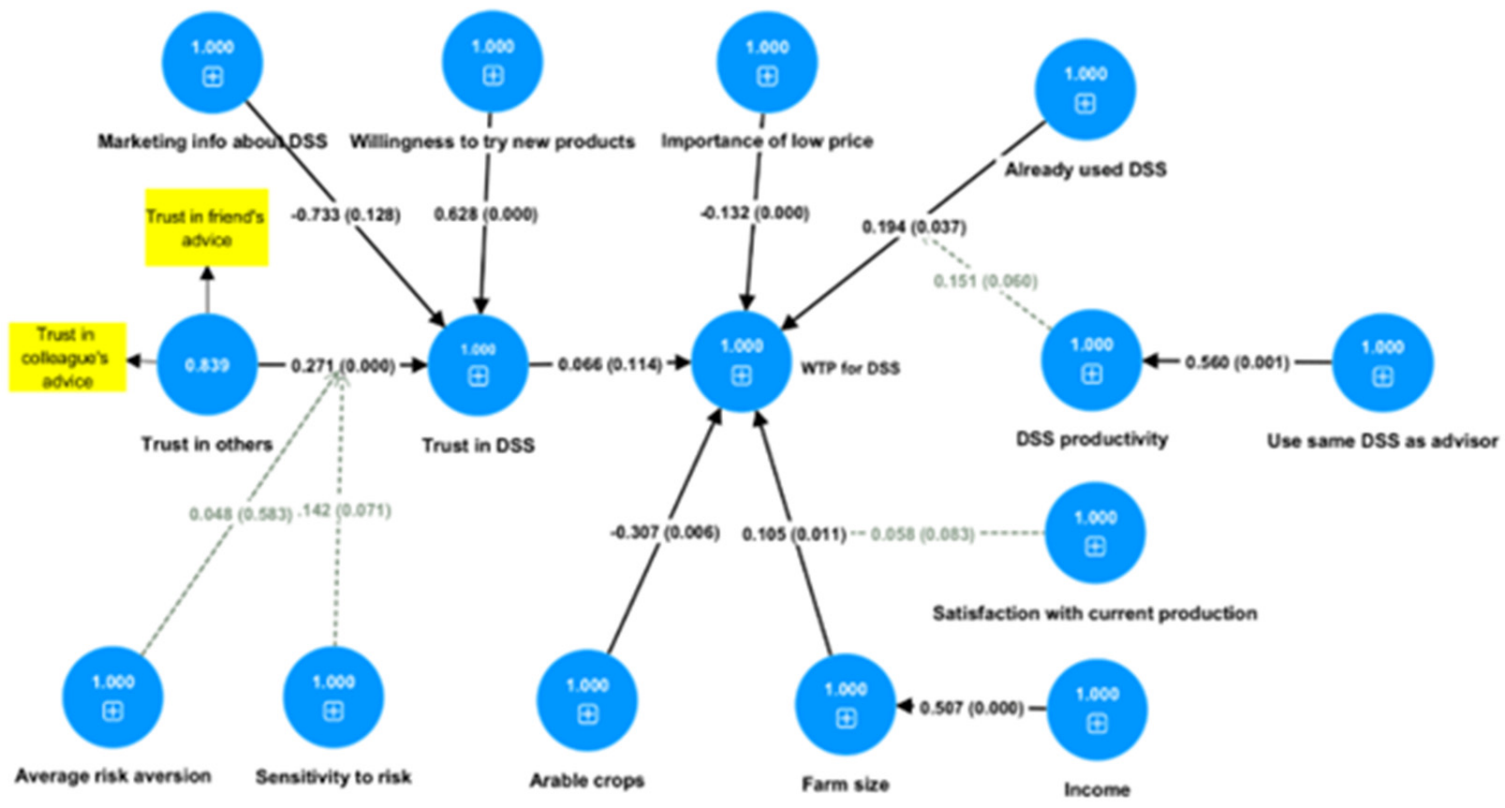
Willingness to pay for DSS model (SEM estimated with SMART PLS). Numbers in circles are model coefficients, numbers on the arrows are path coefficients and numbers in parentheses are p-values.

Figure 2 presents the results of a second model (detailed results are in Appendix C, Table 1 in Extended data) which concerns the monetary valuation of a DSS, measured by the user’s WTP. The model has been estimated as an independent model from the first one, because the decision to use a DSS is almost a necessary condition for the user’s WTP. Overall, the mechanism behind a significant monetary valuation of a DSS by a potential user is a rather complex one. Experience using a DSS is found to be significantly associated with the WTP for it. The effect is further enhanced by the user’s perception that the DSS is efficient, which is positively associated with the use of the same DSS as one’s advisor. Again, a larger farm and a specialisation in arable crop farming significantly contribute to the economic value of the DSS as reflected by the user’s WTP for it. The effect of a larger farm is further enhanced, when farmers are satisfied with their current level of production. Therefore, stability in a farmer’s plans make the farm size effect stronger. Farm size is also positively associated with income, and this is the way in which income is also positively related to the WTP for a DSS. Farmers who are sensitive to the price of a DSS are less willing to pay for it. On the left-hand side of the graph, we find variables which affect the user’s trust in DSS. This is driven mostly by psychological traits, like the farmer’s tendency to trust in others (composed by trust in friends’ and colleagues’ advice) and the farmer’s risk attitude. In this risk attitude-related effect, sensitivity to variations in risk-return trade-offs plays a more significant role than risk aversion. This sensitivity to risk-returns may even be capturing the farmers’ better ability to calculate the risks, thus reacting more rationally to the challenges of their environment. Finally, farmers’ intrinsic willingness to try new products plays a significant role in their trust in a DSS, while marketing sessions have no statistically significant effect, but if there were any effect the sign would be negative. Surprisingly, the farmer’s trust in DSS seems to have only a weak positive effect on their willingness to pay for a DSS.

## Discussion and main conclusions

From our analysis above, the structural equation modelling techniques adopted here uncover a more complex process underlying a potential user’s decision to adopt a DSS and the willingness to pay for it. Furthermore, this process consists of a structure of interdependent determinant factors that related to either the features of the farm, or the farmer’s perceptions and attitudes that align with broader findings in the adoption of digital technologies in agriculture.

Farm size is a significant determinant factor of both adoption and WTP, with larger farms more likely to adopt and willing to pay for DSS. This finding is strongly supported by Dibbern *et al.* (2024) in the adoption of digital technologies as large farms are likely to adopt because they have more resources and enjoy economies of scale. When engaging with larger farms, promotion activities should focus on the benefits derived from DSS use, including productivity gains, environmental sustainability and cost savings which are some of the key drivers of the adoption of digital agricultural technologies ( Dibbern *et al*., 2024). On the other hand, when engaging with smaller farms, promotion activities should focus on eliminating price barriers by offering different pricing strategies. and demonstrating the cost saving benefits derived from their use, such as reduced input costs, increased production through lower input or targeted inputs according to farmers’ needs.

The main crop type grown on the farm is a significant determinant of WTP and adoption. From our analysis, arable crop farmers are less willing to pay to use DSS but more likely to adopt it if they have large farms, reflecting the complex and large-scale operations associated with arable crop farming. On the other hand, small scale arable crop farmers exhibit a higher WTP but are less likely to adopt. They may be aware of the potential benefits but are constrained by their operational scale. When engaging with large-scale arable farmers, promotion should focus on demonstrating the long-term benefits. An estimation on how long it will take to break even and recover their initial investment will reduce the uncertainty and improve WTP. Similarly, when engaging with small scale arable farms, promotion should also focus on reducing adoption costs by availing affordable pricing mechanisms and the development of DSS specifically tailored for small scale operations. Rose *et al*. (2016) suggest that decision support tools must be extensively trialled in the field before they are released to the public. This targeted approach is also recommended by Dibbern *et al*. (2024) to promote the use of digital agricultural technologies. In addition, for farmers who specialise in a single crop, promotion activities should focus on identifying the specific crop/pests of interest and connecting them directly with appropriate systems and/or developers to create them.

Farmer characteristics such as experience and attitudes play a crucial role in WTP and DSS adoption. Farmers with previous experience with DSS have a higher WTP, particularly among those who believe DSSs will improve their productivity and use the same DSSs as their advisors. The study by Bonke *et al*. (2018) show that information, potential for cost reduction and reduced environmental impacts significantly influence the WTP for agricultural applications To improve DSS adoption, developers should provide a platform where veteran and amateur farmers can engage and share success strategies, and unify DSS versions for farm extension officers and farmers for better service delivery.

Farmers appear less willing to pay for relatively low priced DSS. This could reflect a perception among farmers that lower prices are associated with less reliable DSS, or lower levels of support. This highlights the need for transparency between users and developers to understand and justify the underlying cost structure of DSS development. As the ease of use is also a significant determinant of adoption, DSSs must be user centric, clear, logical, and intuitive. This aligns with the findings from Rose *et al*. (2016) that focussing on user centered design and collaborative development can improve the adoption of decision support tools in decision making. Once again, this highlights the need for direct engagement between developers and targeted end users throughout the development of DSS and associated platforms. Developers should also showcase the clarity of the GUI, level of customization, types of visual aids, adaptability to different system platforms, and be open to adapt systems based on feedback to maintain ease of use for farmers.

Based on these insights, we recommend targeted promotion strategies and development among EU farmers ( Dibbern *et al*., 2024). Incorporating behavioural drivers of decision making in various stages of DSS development lifecycle and implementation can greatly improve adoption and use. Specifically, promotion strategies must account for farmers with different farm sizes or experience. Offering a more generalised, free-to-use DSS could serve as a gateway to engagement, building trust and facilitating a transition towards more advanced, targeted pay-per-use systems. Furthermore, relevant case studies demonstrating successful implementations of IPM DSS in practice can be far more effective in building trust than generic marketing campaigns. This approach aligns with Rose *et al*. (2016) that recommendation promotion through leveraging trusted peer networks. In the same vein, understanding farmers’ risk attitudes in relation to adoption of agricultural DSSs can provide valuable insights for marketing and product demonstration events. This is particularly relevant given the findings by Jin *et al*. (2024) which highlight the importance of considering "individual, regional, and cultural forces" in shaping farmers' decisions

## Limitations of the study

Even though this study takes on a unique approach to model the adoption of agricultural DSSs by farmers in pesticide management, it also has several limitations. Firstly, the relatively small sample size from 11 European countries is inadequate to generalize the findings across the entire European farming population and other farmers outside Europe with different farming practices. Furthermore, there is an imbalance in the number of participants per country that can introduce bias. Lastly, as the survey was conducted just before the onset of the COVID pandemic, it may not account for shifts in attitudes or DSS adoption patterns because of the global crisis. In conclusion, these limitations suggest areas for further research on the factors influencing the adoption of DSS in pesticide management, such as using time series data to understand how the decision-making process changes over time and establish causal relationships.

## Ethical approval and consent

The data was collected in accordance with Article 14 of the model grant agreement, and a self-assessment ethics assessment was undertaken prior to surveys and workshops, including the completion of an Ethics Issues Table.

No further ethics approvals were required as all participants were over 18 years old; no sensitive data were collected; the data were pseudonymized; and the analysis was independent of personal data. The surveys were reviewed and overseen by the IPM Decisions Ethics Board, chaired by an independent chair. All relevant activities respected European and national laws, and ethics approval was obtained by relevant institutional and/or national parties as required.

Written informed consent was obtained from all participants.

## Data Availability

Zenodo: Raw (main) dataset for the paper "Decision Support Systems Adoption in Pesticide Management" https://zenodo.org/doi/10.5281/zenodo.11409484 ( Akaka *et al*., 2024a). This project contains the following extended data: Zenodo: Curated dataset for the paper "Decision Support Systems Adoption in Pesticide Management" https://zenodo.org/doi/10.5281/zenodo.10888113 ( Akaka *et al*., 2024b). Zenodo: Appendices for the paper "Decision Support Systems Adoption in Pesticide Management" https://zenodo.org/doi/10.5281/zenodo.11403256 ( Akaka *et al*., 2024c). Data are available under the terms of the Creative Commons Attribution 4.0 International license (CC-BY 4.0).
