# Supplementary material for: Decision Support Systems adoption in pesticide management
Source: Open Res Eur. 2024 Sep 26;4:142. Originally published 2024 Jul 10. [Version 2] doi: 10.12688/openreseurope.17577.2 (PMC11754955; doi:10.12688/openreseurope.17577.2)
Supplement: Peer review history

# Reviewer report: Reviewer response for version 2

**DOI:** 10.21956/openreseurope.20067.r49527

**Published:** 2025-01-23

**Authors:** James A. Turner

**Affiliations:** 1 AgResearch Ltd, Ruakura Research Centre, Hamilton, New Zealand

The paper draws from a cross-country survey of arable crop farmers willingness to adopt (WTA) and willingness to pay (WTP) for Decision Support Systems (DSS) for managing pesticide use. The authors present a structural equation model (SEM) to explore the potential relationship among farm, farmer, DSS and external factors on WTA and WTP for DSS. The study is potentially novel in the analysis of the multitude of factors shaping adoption of DSS, the separate analysis of factors shaping WTA and WTP, and the inclusion of a risk elicitation method to develop a more complete description of risk attitudes. The paper presents useful conclusions and recommendations for supporting increased uptake by arable crop farmers of DSS for improved pesticide management. The paper is well written, clear and easy to understand the methods used and conclusions drawn.

 There are a couple of minor aspects that could be address to further improve the paper: 

 (i)    Strengthening the link between the factors hypothesised to influence WTA (Model) and WTP (Model 2) by including citations against the hypotheses. For example, why is the effect of specialising in a single crop on WTA DSS hypothesised to be stronger for male farmers?

 (ii)    Recognising in the Limitations of the study section the limitation raised by a previous reviewer regarding having insufficient sample size to be able to analyse differences in the influence of factors due to different DSS characteristics and cropping systems. This then clarifies why the paper does not explore these factors.

 Specific comments/edits:

 (i)    There remain a few minor grammatical errors throughout the paper, e.g. p. 4 first paragraph 4th line “…only are some DSS are overwhelmingly complex...”

 (ii)    p. 4 column 1 last paragraph, suggest adding de Oca Munguia and Llewellyn (2020) and other citations to support claim that “a majority of this research mostly identified technological features and user traits…”

 (iii)    p. 4 column 2 first paragraph, suggest recognising that Kuehne et al. (2017) is one of few studies that have considered experience, individual risk attitudes and trust. Also look if there have been other applications of the ADOPT Tool to DSS uptake and cite these

 (iv)    p. 4 column 2 under “Survey to model Adoption decision and WTP”. What is PME? 

 (v)    P. 5 column 1 paragraph 3, please specify what the purpose of the national workshops were? In inviting farmers to attend what was the purpose that was included in the invite. If the purpose of the workshop was about DSS then this may have biased the results toward WTA as farmers are indicating an interest in engaging with DSS by attending the workshop.

 (vi)    P. 6 column 2 second paragraph, 4th line – please clarify what “Its” is

 (vii)    P. 9 third paragraph, line 14, please specify who recommended promotion leverage trusted peer networks: Rose et al. (2016). 

 (viii)    It was an interesting finding that marketing sessions have no statistically significant effect on WTP. The paper then recommends promoting DSS. A sentence or two to clarify how such promotion differs from marketing would address what could be seen as a counter intuitive recommendation given the lack of effect from marketing.

Is the study design appropriate and does the work have academic merit?

Yes

Is the work clearly and accurately presented and does it cite the current literature?

Partly

If applicable, is the statistical analysis and its interpretation appropriate?

Yes

Are all the source data underlying the results available to ensure full reproducibility?

Yes

Are the conclusions drawn adequately supported by the results?

Partly

Are sufficient details of methods and analysis provided to allow replication by others?

Yes

Reviewer Expertise:

agricultural extension, resource economics, agricultural innovation systems, qualitative social research

I confirm that I have read this submission and believe that I have an appropriate level of expertise to confirm that it is of an acceptable scientific standard.

## References

[1] : The Adopters versus the Technology: Which Matters More when Predicting or Explaining Adoption?. *Applied Economic Perspectives and Policy* .2020;42(1) : 10.1002/aepp.13007 80-91 10.1002/aepp.13007

[2] : Predicting farmer uptake of new agricultural practices: A tool for research, extension and policy. *Agricultural Systems* .2017;156: 10.1016/j.agsy.2017.06.007 115-125 10.1016/j.agsy.2017.06.007

# Reviewer report: Reviewer response for version 2

**DOI:** 10.21956/openreseurope.20067.r49092

**Published:** 2025-01-22

**Authors:** Andrea Sciarretta

**Affiliations:** 1 Department of Agricultural, Environmental and Food Sciences, University of Molise, Campobasso, Italy

The paper presented the results of a survey on the adoption of DSS by farmers in several European countries. The work is certainly interesting and considers various factors that may influence farmers' choices. It is a very important topic, because often the adoption of digital tools in agricultural practice represents the real bottleneck, more than their design or effectiveness.

 The most evident weakness seems to me the small sample size examined, an aspect that the Authors partly addressed in the Conclusions. For this reason, some important variables are lost, such as the geographical origin of the respondents (even if a rough division between Med and not-Med countries could be done). In spite of the small sample, the results are clear and provide important insights into the factors influencing farmers' choices. Thus, I consider the paper provide significant contribution to this relevant issue.

 The most significant shortcoming concerns information on the type of crop. Only arable crop is generally mentioned. Were other categories considered that were not significant? Which ones? Some additional information needs to be included, at least to better clarify the arable/non-arable crops considered.

 Nothing is said about the type of DSS proposed to the respondents. Did you present in the project a particular type of DSS? When you discussed the DSS in the workshops, what was the type of application for which was set up?

 In Data analysis, you refer to different panels, without having introduced what these panels represent.

Is the study design appropriate and does the work have academic merit?

Yes

Is the work clearly and accurately presented and does it cite the current literature?

Yes

If applicable, is the statistical analysis and its interpretation appropriate?

Yes

Are all the source data underlying the results available to ensure full reproducibility?

Yes

Are the conclusions drawn adequately supported by the results?

Yes

Are sufficient details of methods and analysis provided to allow replication by others?

Partly

Reviewer Expertise:

Integrated Pest Management, Pest control tools, DSS design for pest control

I confirm that I have read this submission and believe that I have an appropriate level of expertise to confirm that it is of an acceptable scientific standard, however I have significant reservations, as outlined above.

# Reviewer report: Reviewer response for version 2

**DOI:** 10.21956/openreseurope.20067.r44661

**Published:** 2024-10-07

**Authors:** Yiorgos Gadanakis

**Affiliations:** 1 University of Reading, Reading, England, UK

I have now reviewed the updated manuscript and I am happy with all changes and amendments. No further comments from my side

Is the study design appropriate and does the work have academic merit?

Yes

Is the work clearly and accurately presented and does it cite the current literature?

Partly

If applicable, is the statistical analysis and its interpretation appropriate?

Yes

Are all the source data underlying the results available to ensure full reproducibility?

Yes

Are the conclusions drawn adequately supported by the results?

Yes

Are sufficient details of methods and analysis provided to allow replication by others?

Partly

Reviewer Expertise:

Agricultural economics, farm business management, sustainability assessment, co-production of services with stakeholders in the agricultural sector

I confirm that I have read this submission and believe that I have an appropriate level of expertise to confirm that it is of an acceptable scientific standard.

# Reviewer report: Reviewer response for version 1

**DOI:** 10.21956/openreseurope.18993.r42227

**Published:** 2024-08-22

**Authors:** Yiorgos Gadanakis

**Affiliations:** 1 University of Reading, Reading, England, UK

Dear authors, 

 Thank you for the opportunity given to review your work.

**Abstract:** The abstract is well-structured and is communicating effectively the main objectives, the methods used and the key findings of this work.

**Introduction: **The abbreviation DSS is introduced in the abstract, in my opinion, it should also be repeated here, especially when it is presented together with IPM DSS.

 Also, the text will need to carefully be edited since there are some comments left i.e. in the first paragraph the comment (add reference) is shown.

 In page 3, right hand side column 3rd paragraph, there are some references in respect to GUI and its complexity. This section is not well cited and is confusing in terms of the source of the information, Is this conclusions derived from the literature or arguments developed by the authors? Overall, there are sections in the introduction that will need to be reviewed and better discussed in terms of the source of the information and how this is presented in the manuscript. 

 Survey to model Adoption decision and WTP: The sample size is small and not well-balanced across countries participating in the survey. I do appreciate that recruiting farmers is difficult, however, the unbalanced sample size amongst countries will need to be addressed. Could the authors explain this unequal distribution? Also, was the survey pilot tested in all countries or in a specific country? How many farmers were interviewed during the pilot phase? Have there been changes? Was the survey administrated in English, or was it translated to other languages? How was validity of the questions and the questionnaire guaranteed? Has the outbreak of COVID 19 influenced the recruitment of farmers? 

**Descriptive statistics:** "All participants in the Work Package 6-related workshops participated on site to the survey and no responses were excluded." this is probably taken from a deliverable of the project. Please review and edit accordingly

**Data analysis: I**s panel in the following sentence "while the second component involves the difference of choices between panel 4 and 1 and between panel 3 and 2 (with the former difference being loaded twice as much as the latter)." referring to the workshop panels? If so, then which countries? 

**Discussion and main conclusions:** I have found the results of the second model really interesting in terms of future recommendations and the importance of factors such as trust and risk. The discussion is well developed and the narrative is well structured. However, what I would have expected to see here is also a comparison of the findings to similar literature and other studies in the field. Although, trust and risk might have not been incorporated on other studies related to IPM DSS they have been discussed in the field of agriculture. Especially when discussing adoption and and WTP for digital technologies in agriculture. Also, interesting too review here is the characteristics of the DSS that the farmers would prefer or that would optimise adoption and functionality. A very interesting read is the following: [ref 1] and also [ref 2]. In addition, the following could be considered [ref 3] and [ref 4]. I believe that the discussion of the findings for this paper will highly benefit from adding evidenced from the literature and how their findings are compared with others in the sector.

Is the study design appropriate and does the work have academic merit?

Yes

Is the work clearly and accurately presented and does it cite the current literature?

Partly

If applicable, is the statistical analysis and its interpretation appropriate?

Yes

Are all the source data underlying the results available to ensure full reproducibility?

Yes

Are the conclusions drawn adequately supported by the results?

Yes

Are sufficient details of methods and analysis provided to allow replication by others?

Partly

Reviewer Expertise:

Agricultural economics, farm business management, sustainability assessment, co-production of services with stakeholders in the agricultural sector

I confirm that I have read this submission and believe that I have an appropriate level of expertise to confirm that it is of an acceptable scientific standard, however I have significant reservations, as outlined above.

## References

[1] : Main drivers and barriers to the adoption of Digital Agriculture technologies. *Smart Agricultural Technology* .2024;8: 10.1016/j.atech.2024.100459 10.1016/j.atech.2024.100459

[2] : Decision support tools for agriculture: Towards effective design and delivery. *Agricultural Systems* .2016;149: 10.1016/j.agsy.2016.09.009 165-174 10.1016/j.agsy.2016.09.009

[3] : Willingness to pay for smartphone apps facilitating sustainable crop protection. *Agronomy for Sustainable Development* .2018;38(5) : 10.1007/s13593-018-0532-4 10.1007/s13593-018-0532-4

[4] : Analysis of Influencing Factors on Farmers’ Willingness to Pay for the Use of Residential Land Based on Supervised Machine Learning Algorithms. *Land* .2024;13(3) : 10.3390/land13030387 10.3390/land13030387

# Response

**Published:** 2024-09-11

**Authors:** Jotham Jea Akaka

**Affiliations:** Economics, Universitat Jaume I, Castellón de la Plana, Valencian Community, Spain

**Reviewer Comment:**

**Abstract:** The abstract is well-structured and is communicating effectively the main objectives, the methods used and the key findings of this work.

*Author Response: Thanks*

**Introduction: **The abbreviation DSS is introduced in the abstract, in my opinion, it should also be repeated here, especially when it is presented together with IPM DSS.

 Also, the text will need to carefully be edited since there are some comments left i.e. in the first paragraph the comment (add reference) is shown.

*Author Response: Done! Thanks*

**Reviewer Comment:**

 In page 3, right hand side column 3rd paragraph, there are some references in respect to GUI and its complexity. This section is not well cited and is confusing in terms of the source of the information, Is this conclusions derived from the literature or arguments developed by the authors? Overall, there are sections in the introduction that will need to be reviewed and better discussed in terms of the source of the information and how this is presented in the manuscript. 

*Author Response: Done! Thanks*

**Reviewer Comment:**

 Survey to model Adoption decision and WTP: The sample size is small and not well-balanced across countries participating in the survey. I do appreciate that recruiting farmers is difficult, however, the unbalanced sample size amongst countries will need to be addressed. Could the authors explain this unequal distribution?

*Author Response: The population of farmers participating in the survey was mostly in situ sample of stakeholders who were contacted, recruited and accepted to participate in the survey. Given that the teams responsible for each country had different channels of contacting farmers, the resulting sample is unbalanced, but the method employed to model decisions is not affected significantly, given that the analysis has not identified significant differences across countries.*

**Reviewer Comment:**

 Also, was the survey pilot tested in all countries or in a specific country?

*Author Response: No pilot session was possible outside our own research team.*

**Reviewer Comment:**

 How many farmers were interviewed during the pilot phase? Have there been changes? Was the survey administrated in English, or was it translated to other languages?

*Author Response: The survey was administered in each country in the corresponding language.*

**Reviewer Comment:**

 How was validity of the questions and the questionnaire guaranteed?

*Author Response: The in-person response during stakeholder workshops has guaranteed the attention of the respondents, with the monitoring of the conditions under which responses were given and submitted.*

**Reviewer Comment:**

 Has the outbreak of COVID 19 influenced the recruitment of farmers? 

*Author Response: We have commented on this in the limitation section. Thanks.*

**Reviewer Comment:**

**Descriptive statistics:** "All participants in the Work Package 6-related workshops participated on site to the survey and no responses were excluded." this is probably taken from a deliverable of the project. Please review and edit accordingly

*Author Response: Done! Thanks*

**Reviewer Comment:**

**Data analysis: I**s panel in the following sentence "while the second component involves the difference of choices between panel 4 and 1 and between panel 3 and 2 (with the former difference being loaded twice as much as the latter)." referring to the workshop panels? If so, then which countries? 

*Author Response:*

*Clarification: “Panel” refers to a specific instrument for risk attitude elicitation, based on a series of lottery panels (See Sabater-Grande and Georgantzis, 2002), not the usual term.*

**Reviewer Comment:**

**Discussion and main conclusions:** I have found the results of the second model really interesting in terms of future recommendations and the importance of factors such as trust and risk. The discussion is well developed and the narrative is well structured. However, what I would have expected to see here is also a comparison of the findings to similar literature and other studies in the field. Although, trust and risk might have not been incorporated on other studies related to IPM DSS they have been discussed in the field of agriculture. Especially when discussing adoption and and WTP for digital technologies in agriculture. Also, interesting too review here is the characteristics of the DSS that the farmers would prefer or that would optimise adoption and functionality. A very interesting read is the following: [ref 1] and also [ref 2]. In addition, the following could be considered [ref 3] and [ref 4]. I believe that the discussion of the findings for this paper will highly benefit from adding evidenced from the literature and how their findings are compared with others in the sector.

*Author Response:  Discussion and conclusions have been re-written based on your suggestions above. We have included the references you suggested.*

# Reviewer report: Reviewer response for version 1

**DOI:** 10.21956/openreseurope.18993.r42225

**Published:** 2024-08-19

**Authors:** David H Gent

**Affiliations:** 1 Forage Seed and Cereal Research Unit, US Department of Agriculture-Agricultural Research Service, Corvallis, OR, USA

The manuscript by Akaka and colleagues uses a survey of farmers in Europe to fit structural equation models that describe farmers' willingness to pay and willingness to adopt decision support-systems for pest management decision making. The research appears to have been conducted carefully. The analyses are novel in that the authors recognize the multidimensional nature of adoption and have used quantitative methods to model the adoption process. The corpus of the work makes contributions to understanding the situated reasons why decision support systems may or may not be adopted and used. With minor revision, the manuscript could be accepted.

 A limitation of the current analysis is the relatively small sample size. Given this, did the authors attempt any analyses to check the robustness of the SEMs? My concern is that that results may be idiosyncratic to the specific population of respondents and difficult to generalize. This impacts one of the main conclusions that the “structure of interdependent determinant factors ...are robust to country differences”. I find this statement to be a hypothesis that requires testing to be confirmed.

 Related the small sample sizes, the cropping systems appear to be quite diverse. It would be useful for the reader to understand the relative value of the crops that were grown, as empirical studies and theory suggests that adoption of decision support systems is sensitive to asymmetry in the costs of type I and type II errors. In this sense, the authors could expand the discussion to consider if certain crops are better suited to decision-support systems based not on the type of crop (ie, arable) but other factors that are causal. For instance, are arable crops well situated simply because they are arable crops or because of the risk-reward ratio for the specific decision-support systems considered?

 The concept of risk reduction from using a decision-support system is underdeveloped in the manuscript.

 It would have been useful for the reader to know which specific decision-support systems respondents had used. If this information was collected, I would highly recommend including a summary in the body of the manuscript and in the associated raw data.

 The statement at the end of the fourth paragraph on page 3 is quite strong considering the multidimensional nature of adoption. A series of citations are needed to support this assertion.  

 A careful review of the manuscript is needed. See the first paragraph for an example of an oversight or typographical error.

Is the study design appropriate and does the work have academic merit?

Yes

Is the work clearly and accurately presented and does it cite the current literature?

Yes

If applicable, is the statistical analysis and its interpretation appropriate?

Yes

Are all the source data underlying the results available to ensure full reproducibility?

Yes

Are the conclusions drawn adequately supported by the results?

Yes

Are sufficient details of methods and analysis provided to allow replication by others?

Yes

Reviewer Expertise:

Plant disease epidemiology, risk analysis, decision support

I confirm that I have read this submission and believe that I have an appropriate level of expertise to confirm that it is of an acceptable scientific standard, however I have significant reservations, as outlined above.

# Response

**Published:** 2024-09-11

**Authors:** Jotham Jea Akaka

**Affiliations:** Economics, Universitat Jaume I, Castellón de la Plana, Valencian Community, Spain

The manuscript by Akaka and colleagues uses a survey of farmers in Europe to fit structural equation models that describe farmers' willingness to pay and willingness to adopt decision support-systems for pest management decision making. The research appears to have been conducted carefully. The analyses are novel in that the authors recognize the multidimensional nature of adoption and have used quantitative methods to model the adoption process. The corpus of the work makes contributions to understanding the situated reasons why decision support systems may or may not be adopted and used. With minor revision, the manuscript could be accepted.

 A limitation of the current analysis is the relatively small sample size. Given this, did the authors attempt any analyses to check the robustness of the SEMs?

 Response: The small sample size is because most of the data were collected in situ during workshops with stakeholders. This is now discussed in the limitation section of our manuscript. While being aware of the small size of our sample, we believe that implementing the survey in such a personalized way increased the quality of the responses and the attention paid by the respondents.

 My concern is that that results may be idiosyncratic to the specific population of respondents and difficult to generalize. This impacts one of the main conclusions that the “structure of interdependent determinant factors … are robust to country differences”. I find this statement to be a hypothesis that requires testing to be confirmed.  

 Response: This comment has been removed to avoid confusion. It was meant to refer to the absence of country effects in our estimation results. We clearly mention this in the absence of the term robustness.

 Related the small sample sizes, the cropping systems appear to be quite diverse. It would be useful for the reader to understand the relative value of the crops that were grown, as empirical studies and theory suggests that adoption of decision support systems is sensitive to asymmetry in the costs of type I and type II errors. In this sense, the authors could expand the discussion to consider if certain crops are better suited to decision-support systems based not on the type of crop (ie, arable) but other factors that are causal. For instance, are arable crops well situated simply because they are arable crops or because of the risk-reward ratio for the specific decision-support systems considered?

 Response: The dataset does not permit identification of or clustering by the DSS used. Furthermore, the small sample size does not allow to segment the sample according to a more detailed breakdown by crop, because this would lead to excessively small and uneven subsamples.

 The concept of risk reduction from using a decision-support system is underdeveloped in the manuscript.

 Response: Given the lack of DSS specificity, our paper has not attempted to make generalisations on issues (like the reduction of risk) which vary across different DSS and use types. In fact, our reference to risk relates more to the farmers’ personal risk attitudes than the objective risks involved in the decisions.

 It would have been useful for the reader to know which specific decision-support systems respondents had used. If this information was collected, I would highly recommend including a summary in the body of the manuscript and in the associated raw data.

 Response: Indeed. But as we mentioned above, this type of data is not available. The DSS used would lead to isolated cases, more than user clusters for statistical analysis and modelling.

 The statement at the end of the fourth paragraph on page 3 is quite strong considering the multidimensional nature of adoption. A series of citations are needed to support this assertion.  Response: We have addressed this by adding a series of citations

 A careful review of the manuscript is needed. See the first paragraph for an example of an oversight or typographical error.

 Response: Thank you! We have done this and hope to have produced a flawless manuscript.
